# The Newest Vital Sign: Frequently Asked Questions

**DOI:** 10.3928/24748307-20180530-02

**Published:** 2018-07-11

**Authors:** Barry D. Weiss

The Newest Vital Sign (NVS) is one of the most widely used health literacy screening instruments (Shealy & Threatt, 2016; Weiss et al., 2005). The original version of the NVS was developed in English and Spanish and validated in the United States for identifying people with limited health literacy skills (Weiss et al., 2005). Since then, the NVS has been adapted and validated for use in other languages and countries, including the United Kingdom (Rowlands et al., 2013), the Netherlands (Fransen et al., 2014), Japan (Kogure et al., 2014), Italy (Capecchi, Guazzini, Lorini, Santomauro, & Bonaccorsi, 2015), Kuwait (Al-Abdulrazzaqa, Al-Haddadb, AbdulRasoula, Al-Basarib, & Al-Taiara, 2015), Brazil (Rodrigues, de Andrade, González, Birolim & Mesas, 2017), China (Xue et al., 2018), and Canada (Mansfield, Wahba, Gillis, & Weiss, 2018). It has also been adapted for administration in American Sign Language (McKee et al., 2015).

To administer the NVS, a person is presented with a nutrition label from a container of ice cream and asked six questions about the label. Correct responses require the ability to identify and interpret basic text and perform simple mathematical computations.

The assessment takes 2 to 3 minutes (Eubanks et al., 2017; Johnson & Weiss, 2008; Welch, VanGeest, & Caskey, 2011), and the probability of a person having limited health literacy is estimated by counting how many of the six questions are answered correctly. With 0 to 1 correct answers, people are scored as having a high likelihood (50% or more) of limited health literacy. With 2 to 3 correct answers, a person has a possibility of limited health literacy. A score of 4 to 6 almost always indicates adequate health literacy using the Test of Functional Health Literacy in Adults as the reference standard (Weiss et al., 2005).

As lead author on the article (Weiss et al., 2005) that reported the original development and validation of the NVS, I receive inquiries regularly asking about proper methods for administration and interpretation from investigators planning to use it in their work. In this article, I list frequently asked questions and provide answers based on the original validation study (Weiss et al., 2005), firsthand administration of the tool, and published reports and communications from investigators around the world who have used the NVS.

## Question 1

Do I need to obtain permission to use the NVS in clinical settings or research projects?

No. Anyone is free to use the NVS in their clinical or research work at no cost. No permission is required.

## Question 2

Can I give the nutrition label and questions to patients or research participants, have them read the six questions on their own, and have them write down their answers, rather than reading the questions to them and having them answer out loud?

No. The NVS was developed as an interviewer-administered health literacy assessment. Asking patients or research participants to read and answer the questions on their own (i.e., self-administration) would add a level of complexity (i.e., the need to read and understand the questions that would otherwise be read to them) that would threaten the validity of the assessment. Similarly, having people write down answers would add a level of complexity (i.e., the need to write) that was not addressed in validating the NVS.

There have been at least two studies of self-administration and both had similar findings. One study involved adolescents in school (L. A. Linnebur & Linnebur, 2018) and the other involved patients in a low-income, primary care setting setting (Warren-Findlow, 2014). In both studies, only about one-half of the participants were able to complete the assessment and there was no comparison with interviewer administration. Thus, there is no evidence that the instrument yields valid results when self-administrated as a paper-and-pencil assessment.

## Question 3

Can an interviewer administer the NVS to groups of patients or research participants, rather than administering it one-on-one?

No. The NVS has not been validated for group administration. Group administration has the potential to cause embarrassment for people who have difficulty answering the questions in the company of other people. This, in turn, could cause anxiety, impair their performance, and lead to in inaccurate results.

## Question 4

What do I say when participants seem to flounder and say they can't answer one or more of the questions?

Don't do anything to make a participant feel like they are inadequate or having trouble. Just say something like, “That's fine. Let's just go on to the next question.” The unanswered question gets a score of 0.

## Question 5

What do I say when participants ask “How am I doing?” or “Did I get that question right?”

Just say something like, “You are doing fine. Now let's go on to the next question.”

## Question 6

If a patient or research participant asks for pencil and paper to aid in math calculations, can I provide them?

Yes. That is acceptable.

## Question 7

Can I give a calculator to participants if they ask for one?

Yes. Based on one study (Miser, Wallace, & Rayan, 2013), it appears that use of a calculator versus no calculator does not change the results of the NVS assessment; however, you should not spontaneously offer a calculator. You should only provide a calculator if a participant asks for one.

## Question 8

Can I administer the NVS by computer?

Yes, with caveats. Until recently, the answer to this question would have been no. However, a recent project, led by the Bureau of Nutritional Sciences of Health Canada, developed an NVS module suitable for computer administration (Mansfield, Wahba, Gillis, & Weiss, 2018). The module uses a visual presentation with voice-over narration that provides participants with visual and oral instructions, so they do not have to read anything. There are two version of the module (one in English and one in French) and the nutrition label is modeled after the nutrition labels used in Canada.

When studied in a diverse population, computer administration and standard one-on-one interviewer administration performed similarly. Only 8% of 222 participants scored in a different health literacy category (low health literacy likely versus low health literacy possible versus adequate health literacy) on computer versus interviewer administration. More importantly, only 3 (1%) of the 222 participants were scored as having adequate health literacy on one version when the other version scored them as low health literacy likely or possible.

However, for this approach to be effective and valid, a lengthy development process was necessary, including extensive user testing along with development and validation of multiple-choice distractor responses for use in the narrated module. Practitioners interested in using or developing a module for computer administration should consult the procedures used by Health Canada (Mansfield, Wahba, Gillis, & Weiss, 2018)

## Question 9

Can I translate the NVS into other languages?

Yes, but several issues need to be taken into consideration. The original NVS was developed and validated in English and Spanish and was based on the nutrition labels used in the United States. Nutrition labels are often formatted differently and have different content in other countries. When people look at unfamiliar nutrition labels, answering the NVS questions may be more difficult than when they answer the questions using one that is familiar, thus jeopardizing the results of the NVS assessment. Furthermore, using the NVS in another language or in another county is not simply a matter of translation. Although the NVS has been successfully adapted for use in other countries and in languages, these adaptions all involved extensive focus group testing and cognitive interviews to assure accurate translations and cultural appropriateness, in addition to validation studies that compared the results of health literacy assessments with the translated NVS to assessments using other health literacy instruments.

Even for adapting the original American English version for use in other English-speaking countries (i.e., The United Kingdom and Canada), an extensive process and validation effort was required (Mansfield, Wahba, Gillis, & Weiss, 2018; Rowlands et al., 2013). Practitioners interested in translating and adapting the NVS for use in other countries and languages should refer to reports of those validation processes to see what was involved.

## Question 10

Do I need any special permission if I'm creating a new version of the NVS (i.e., translating it into other languages or developing computer modules)?

Yes. Permission must be obtained from the copyright holder before undertaking adaption of the NVS into a language or format other than the original. Information about how to obtain this permission (which is granted at no cost) can be obtained from the author of this article.
